# Right heart and left atrial strain to differentiate cardiac amyloidosis and Fabry disease

**DOI:** 10.1038/s41598-024-52890-y

**Published:** 2024-01-30

**Authors:** Isabel Mattig, Tilman Steudel, Karin Klingel, Gina Barzen, David Frumkin, Sebastian Spethmann, Elena Romero Dorta, Karl Stangl, Bettina Heidecker, Ulf Landmesser, Fabian Knebel, Sima Canaan-Kühl, Katrin Hahn, Anna Brand

**Affiliations:** 1 Deutsches Herzzentrum der Charité, Department of Cardiology, Angiology and Intensive Care Medicine, Campus Charité Mitte, Chariteplatz 1, 10117 Berlin, Germany; 2 Charité – Universitätsmedizin Berlin, corporate member of Freie Universität Berlin and Humboldt-Universität zu Berlin, Charitéplatz 1, 10117 Berlin, Germany; 3 Amyloidosis Center Charité Berlin (ACCB), Charité – Universitätsmedizin Berlin, Berlin, Germany; 4 DZHK (German Centre for Cardiovascular Research), Partner Site Berlin, Berlin, Germany; 5 Berlin Institute of Health at Charité – Universitätsmedizin Berlin, BIH Biomedical Innovation Academy, Berlin, Germany; 6 Cardiopathology, Institute for Pathology and Neuropathology, University Hospital Tuebingen, Tuebingen, Germany; 7 Deutsches Herzzentrum der Charité, Department of Cardiology, Angiology and Intensive Care Medicine, Campus Benjamin Franklin, Berlin, Germany; 8 Sana Klinikum Lichtenberg, Innere Medizin II: Schwerpunkt Kardiologie, Berlin, Germany; 9 Charité – Universitätsmedizin Berlin, corporate member of Freie Universität Berlin and Humboldt-Universität zu Berlin, Medizinische Klinik Mit Schwerpunkt Nephrologie Und Internistische Intensivmedizin, Fabry Zentrum, Zentrum für Seltene Nierenerkrankungen (CeRKiD), Campus Charité Mitte, Berlin, Germany; 10 Charité – Universitätsmedizin Berlin, corporate member of Freie Universität Berlin and Humboldt-Universität zu Berlin, Klinik für Neurologie und Experimentelle Neurologie, Berlin, Germany

**Keywords:** Cardiology, Diseases

## Abstract

Echocardiographic differentiation of cardiac amyloidosis (CA) and Fabry disease (FD) is often challenging using standard echocardiographic parameters. We retrospectively analyzed the diagnostic accuracy of right heart and left atrial strain parameters to discriminate CA from FD using receiver operating characteristic curve analyses and logistic regression models. A total of 47 FD and 88 CA patients with left ventricular wall thickening were analyzed. The comparison of both cardiomyopathies revealed significantly reduced global and free wall longitudinal right ventricular strain (RVS; global RVS: CA − 13 ± 4%, *n* = 67, vs. FD − 18 ± 4%, *n* = 39, *p* < 0.001) as well as right atrial strain (RAS; reservoir RAS: CA 12 ± 8%, *n* = 70, vs. FD 26 ± 9%, *n* = 40, *p* < 0.001) and left atrial strain (LAS) in CA patients. Individually, global RVS as well as phasic LAS and RAS showed the highest diagnostic accuracy to distinguish CA and FD. The best diagnostic accuracy was achieved by combining the age, basal RV diameter, global RVS, and reservoir and conduit RAS (area under the curve 0.96 [95% CI 0.90–1.00]). Differential echocardiographic diagnostic work-up of patients with suspected CA or FD can be improved by integrating structural and functional parameters of the right heart and the left atrium.

**Trial registration**: DRKS00027403.

## Introduction

Left ventricular (LV) wall thickness ≥ 12 mm is a common finding in various cardiomyopathies such as hypertrophic cardiomyopathy, cardiac amyloidosis (CA), Fabry disease (FD), and hypertensive heart disease^1^. Especially in CA and FD, diagnosis is often challenging using standard echocardiographic imaging with a considerable delay of confirmation of both diseases^2,3^ impeding access to specific therapeutic approaches that have recently become available.

CA is frequently underdiagnosed in patients with heart failure (HF), particularly in HF with preserved ejection fraction (HFpEF)^4^. It is characterized by an extracellular accumulation of mis-folded proteins including hereditary (ATTRv) or wild type transthyretin (ATTRwt) and monoclonal immunoglobulin light chains (AL) as the most common forms^5^. As recommended by the European Society of Cardiology (ESC), CA should be suspected in patients with LV wall thickness ≥ 12 mm and a variety of additional findings such as heart failure, reduced longitudinal LV strain (LVS), and present apical sparing pattern in echocardiographic assessment^5^. Additional to these typical findings, echocardiographic studies in CA patients revealed a characteristic myocardial texture known as granular sparkling, a reduced LV systolic and diastolic function, an enlargement of the left atrium (LA), a reduction of right ventricular (RV) function as well as pericardial effusion^6,7^. However, these echocardiographic red flags may be of limited diagnostic value to discriminate specific etiologies while reductions of LA strain may be of higher accuracy to diagnose CA in patients with increased LV wall thickness^8^. Prognosis is often poor depending on the amyloid type and disease stage^5,9^ but can be improved by specific therapeutic approaches^10^.

FD is a rare X-linked lysosomal storage disorder with minimal or absent α-galactosidase A (GLA) activity associated with a reduced life-expectancy, mostly due to cardiovascular events^11^. Intracellular cardiac deposition of globotriaosylceramide (Gb3) leads to a staged progression of FD with a silent phase, including a microvascular stage, followed by a detectable accumulation stage, and a clinical phase with inflammation, hypertrophy, and fibrosis^12^. Typical echocardiographic characteristics of FD comprise prominent papillary muscles, reduced basal-lateral LVS and diastolic dysfunction beside the thickened LV wall^13^.

Both cardiomyopathies require a specific treatment to prevent further cardiac damage. Hussain et al.^14^ observed a significant increase in life expectancy of CA patients with Tafamidis therapy. Enzyme replacement therapy in FD led to a reduction of LV mass and reduced severe events^15,16^. Moreover, also chaperon therapy was associated with a reduced or at least stabilized LV mass, especially in case of early treatment initiation^17–19^. Therefore, a timely and correct diagnosis enabling early treatment of both cardiomyopathies is essential for lifetime management of CA and FD patients.

CA red flags as reported by the ESC and echocardiographic characteristics of FD as proposed in multiple studies commonly concentrate on the assessment of the LV^5,13,20^. To the best of our knowledge, the present retrospective analysis is the first study with focus on the morphology and function of the RV, right atrium (RA), and LA to increase the diagnostic accuracy in CA and FD patients. The aim of the study is to evaluate and compare the diagnostic value of diverse echocardiographic parameters as well as RV, RA, and LA mechanics to discriminate both cardiomyopathies.

## Methods

### Study design

FD and CA patients with an age ≥ 18 years treated at the Charité – Universitätsmedizin Berlin, Germany from 2019 to 2022 were retrospectively screened for study participation (German clinical trials registry DRKS00027403). Diagnosis of FD was confirmed by genetic testing of GLA mutation according to current recommendations^20^. CA was assessed by laboratory measurements, echocardiography, scintigraphy, magnetic resonance imaging and/or biopsy including immunohistochemical amyloidosis subtyping as proposed by the ESC^5^. Patients without cardiac involvement of disease or with insufficient echocardiographic image quality to perform RV strain (RVS) or RA strain (RAS) and LA strain (LAS) analysis were excluded. The study was performed in accordance with the Declaration of Helsinki and approved by the institutional ethics committee of the Charité – Universitätsmedizin Berlin, Germany (EA4/224/21 and EA1/014/20)^21^. Requirement for informed consent was waived by the ethics committee of the Charité – Universitätsmedizin Berlin due to the retrospective design of the study^21^.

### Echocardiographic assessment

**Figure 1:**
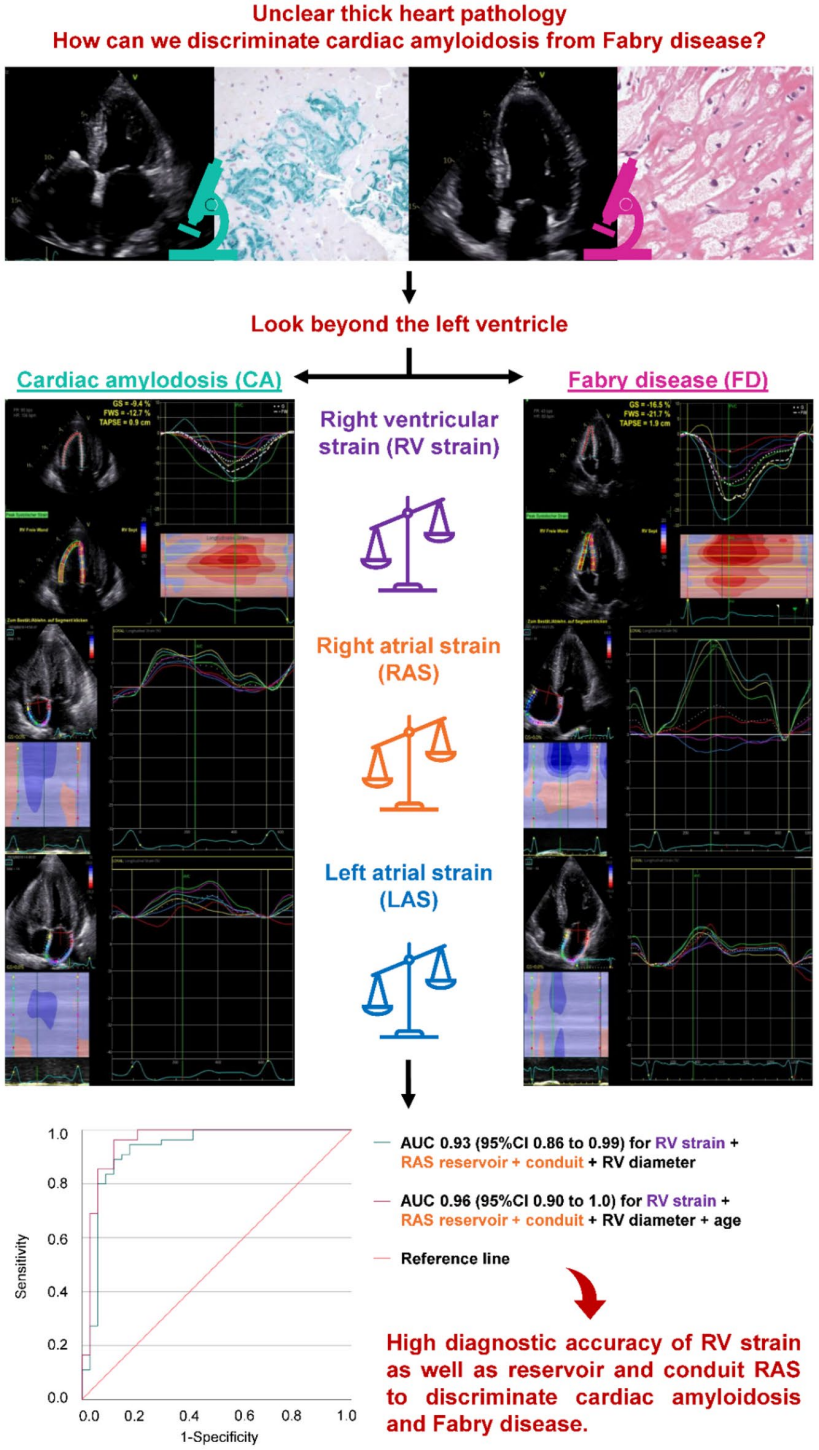
Assessment of longitudinal right ventricular strain, phasic right and left atrial strain in in cardiac amyloidosis and Fabry disease as well as receiver operating characteristic (ROC) curve analysis to differentiate between both cardiomyopathies.

Echocardiographic examinations were performed according to the guidelines of the American Society of Echocardiography (ASE) and the European Association of Cardiovascular Imaging (EACVI) using a GE healthcare Vivid E9 or E95 and a M5S 1.5–4.5 MHz transducer (GE Vingmed, Horton, Norway)^22–24^. Retrospective assessment comprised parameters of cardiac morphology and function listed in Tables 1 and 2 as well as Tables S1 and S2 in the supplement. 2D speckle tracking echocardiography (2D STE) was performed offline (EchoPAC PC, GE Vingmed, Horton, Norway) analyzing echo loops with frame rates between 60 and 90 to assess longitudinal RVS and phasic RAS as well as LAS (Fig. 1). Strain analyses were conducted three times in each patient to calculate the arithmetic mean^25^. Longitudinal RVS including global, free wall and six individual RV segments was performed from an RV-focused apical four-chamber view as recommended by the EACVI^25,26^. Delta RVS was calculated as the difference between free wall longitudinal RVS and global longitudinal RVS. The RVS ratio was calculated as the ratio of basal and mid ventricular septal region to basal and mid ventricular free wall region. The endocardial border was manually traced (RAS and LAS) or automatically reached with a three-point approach and manually adapted afterwards (RVS). The region of interest was determined semi-automatically by the EchoPAC software and manually corrected where indicated. RAS and LAS were calculated from the apical four-chamber view using a QRS-triggered strain curve^25^. Atrial reservoir strain (ASr) was measured at the highest average value of the strain curve^25^. Atrial conduit strain (AScd) during the passive LV filling was calculated as the difference of atrial strain value at the onset of the P wave minus ASr, or as (negative) strain at end-diastole in patients with atrial fibrillation^25^. Atrial contraction strain (ASct), i. e. peak atrial contraction strain, was assessed by the difference of maximum atrial contraction and atrial strain at the onset of the P wave and was not measured in atrial fibrillation^25^. We previously reported on inter- and intra-observer variability for strain assessment which showed very good to excellent intraclass correlations^27–29^.

**Table 1:** Baseline characteristics.

	Cardiac amyloidosis (*n* = 88)	Fabry disease (*n* = 47)	*p*-value
Female, n (%)	21 (21)	22 (47)	0.002
Age at time of echocardiography, years (IQR)	80 (72–84)	54 (43–60)	< 0.001
BMI, kg/m^2^ ± SD	25 ± 3	25 ± 4, *n* = 45	0.707
NYHA class	*n* = 88	*n* = 39	< 0.001
I, n (%)	6 (7)	27 (57)	
II, n (%)	16 (18)	8 (17)	
III, n (%)	28 (32)	4 (9)	
IV, n (%)	2 (2)	0 (0)	
Not assessed, n (%)	36 (41)	8 (17)	
LVEF, % ± SD	50 ± 9	57 ± 8	< 0.001
IVSd, mm ± SD	18 ± 4	15 ± 4	< 0.001
Valvular heart disease			
MR ≥ 2 + , n (%)	17 (19)	6 (13)	0.335
MS ≥ 2 + , n (%)	1 (1)	0 (0)	0.463
AR ≥ 2 + , n (%)	11 (13)	2 (4)	0.122
AS ≥ 2 + , n (%)	2 (2)	0 (0)	0.298
TR ≥ 2 + , n (%)	27 (31)	5 (10)	0.009
TS ≥ 2 + , n (%)	0 (0)	0 (0), *n* = 46	Not assessed
PR ≥ 2 + , n (%)	5 (6)	0 (0)	0.096
PS ≥ 2 + , n (%)	0 (0)	0 (0)	Not assessed
Pericardial effusion, n (%)	22 (25)	4 (9)	0.021
NT-proBNP, ng/l (IQR)	2861 (1460–5434)	186 (71–814)	0.005
Serum creatinine, mg/dl (IQR)	1.1 (0.9–1.5)	0.9 (0.7–1.3)	0.001
Coronary artery disease, n (%)	29 (33), *n* = 83	5 (11), *n* = 46	0.003
Pacemaker, n (%)	9 (10), *n* = 80	2 (4), *n* = 46	0.186
Cardioverter defibrillator, n (%)	2 (2), *n* = 80	4 (9), *n* = 46	0.116
Atrial fibrillation, n (%)	42 (48), *n* = 82	6 (13), *n* = 46	< 0.001
Arterial hypertension, n (%)	65 (74), *n* = 81	19 (40), *n* = 46	< 0.001
Diabetes mellitus, n (%)	15 (17), *n* = 81	3 (6), *n* = 46	0.062
Chronic obstructive pulmonary disease, n (%)	4 (5), *n* = 80	3 (6), *n* = 46	0.720
Specific medical treatment, n (%)	39 (44); tafamidis 39 (44)	30 (64); agalsidase alfa 8 (17), agalsidase beta 6 (13), chaperone 14 (30), unknown type 2 (4)	0.036

Continuous variables are shown as mean ± standard deviation (normally distributed) or median and interquartile ranges (IQR, not normally distributed), categorical variables are given as absolute number with percentages

*BMI* Body mass index, *NYHA class* New York Heart Association Class, *LVEF* Left ventricular ejection fraction, *IVSd* Interventricular septal diameter at end-diastole, *MR ≥ 2 +* Moderate or severe mitral regurgitation, *MS ≥ 2 +* Moderate or severe mitral stenosis, *AR ≥ 2 +* Moderate or severe aortic regurgitation, *AS ≥ 2 +* Moderate or severe aortic stenosis, *TR ≥ 2 +* Moderate or severe tricuspid regurgitation, *TS ≥ 2 +* Moderate or severe tricuspid stenosis, *PR ≥ 2 +* moderate or severe pulmonary regurgitation, *PS ≥ 2 +* Moderate or severe pulmonary stenosis, *NT-proBNP* N-terminal pro brain natriuretic peptide.

**Table 2:** Speckle tracking echocardiography of the right ventricle.

	Cardiac amyloidosis (*n* = 67)	Fabry disease (*n* = 39)	*p*-value
Global longitudinal RVS, % ± SD	− 13 ± 4	− 18 ± 4	< 0.001
Free wall longitudinal RVS, % ± SD	− 16 ± 5	− 21 ± 5	< 0.001
Delta RVS, % ± SD	− 4 ± 3	− 4 ± 2	0.594
RVS ratio, % (IQR)	0.31 (0.19–0.53)	0.62 (0.50–0.75)	< 0.001

Continuous variables are shown as mean ± standard deviation (normally distributed) or median and interquartile ranges (IQR, not normally distributed)

*RVS* Right ventricular strain, *Delta RVS* Difference between free wall longitudinal RVS and global longitudinal RVS, *RVS ratio* Ratio of basal and mid ventricular septal region to basal and mid ventricular free wall region.

### Statistical analysis

Statistical assessment was performed with SPSS Statistics version 28 for Windows (IBM Corporation, New York, NY, USA). Variables are listed in percentages (categorical variables), median with 25th and 75th percentile or mean with standard deviation (continuous variables, depending on skewness, uniform per variable for a better comparison). Categorical variables were analysed by Chi squared test. Statistical analysis of continuous variables was performed using a t-test assuming a normal distribution or a Mann–Whitney-U-test in case of not normally distributed parameters. A *p*-value of < 0.05 was defined as statistically significant. Diagnostic accuracy was evaluated using receiver operating characteristic (ROC) curve analysis with area under the curve (AUC) and confidence intervals (CI). Logistic regression and subsequently ROC curve analysis were performed to include covariates. Parameters were selected for the model according to their clinical relevance, AUC value, Nagelkerke`s R^2^ and if there was a significant difference between the two groups.

## Results

We retrospectively screened 54 FD and 126 CA patients treated at the Charité – Universitätsmedizin Berlin, Germany for study participation. Echocardiographic assessment was performed in 135 patients due to the exclusion of patients with lack of RV-focused echocardiographic images or insufficient quality for 2D STE (FD *n* = 7, CA *n* = 38). Baseline characteristics are presented in Table 1. Patients with FD were significantly younger, showed a higher proportion of women and less symptom burden than subjects with CA. Mean interventricular septal thickness at end-diastole was 18 ± 4 mm in CA and 15 ± 4 mm in FD patients. Patients with CA had the following subtypes of amyloidosis: 62 patients (70%) ATTRwt, 18 patients (21%) ATTRv, 6 patients (7%) AL, and one patient (1%) AA CA. FD patients comprised 24 classic (51%) and 19 late-onset mutations (40%) as well as 4 variants of unknown significance (9%).

### Standard echocardiographic parameters

Standard echocardiographic parameters are listed in Table S1. In comparison of both cardiomyopathies, CA patients presented a more dilated RA, LA, and RV. The RV wall and interatrial septum were thicker and RV function lower in patients with CA, as measured by tricuspid plane systolic excursion (TAPSE), systolic tricuspid annular velocity (RV-Sʹ) and right ventricular fractional area change (RVFAC).

### Speckle tracking echocardiography

**Figure 2:**
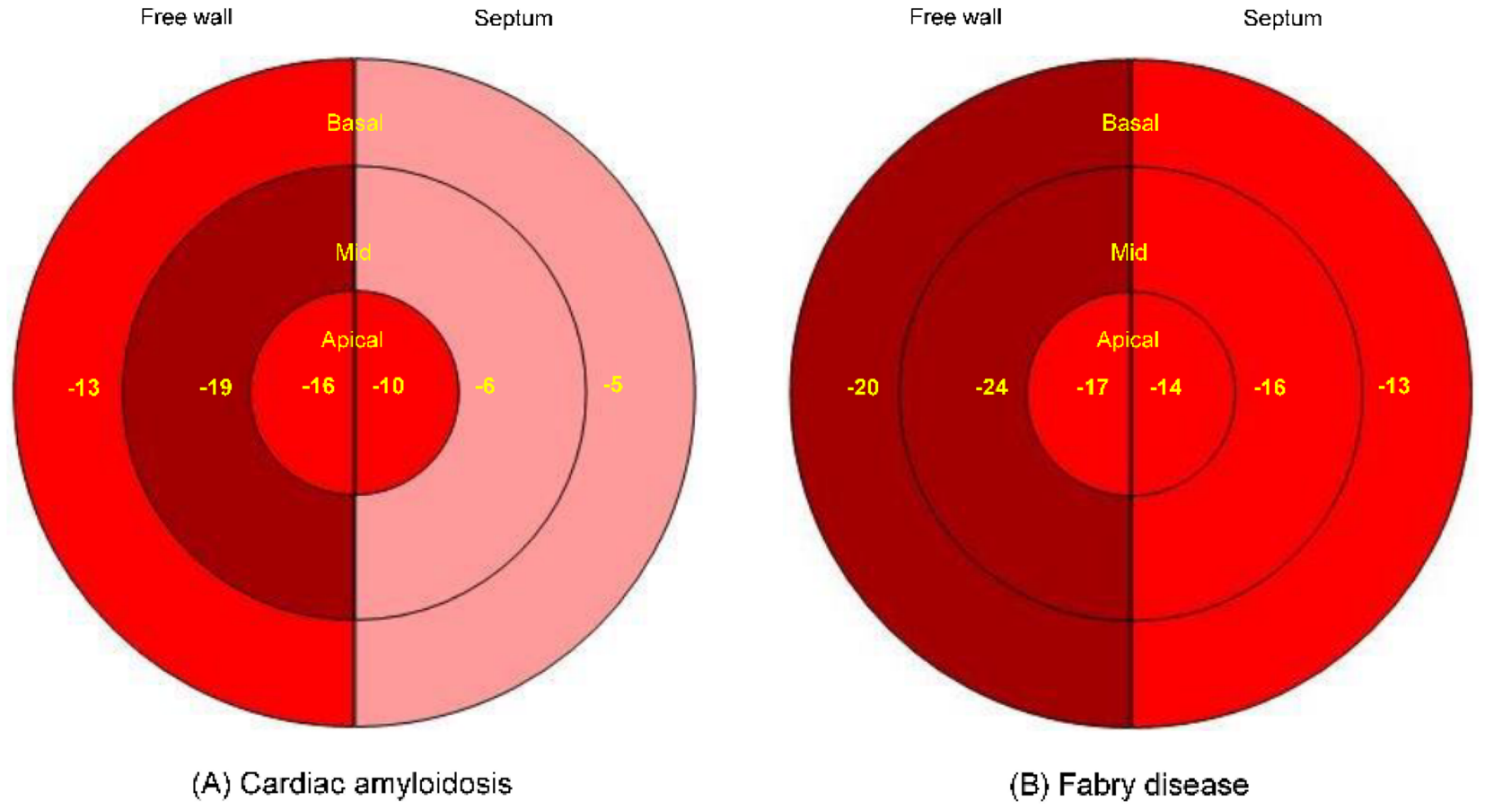
Bull's eye plots of the median right ventricular strain including the free wall (left side), septum (right side) and the apical, mid and basal segments (from the inside out) in cardiac amyloidosis (**A**) and Fabry disease (**B**).

Global and free wall longitudinal RVS were reduced in patients with CA (*n* = 67) compared to FD (*n* = 39), whereas delta RVS was not different between the two groups (Table 2). Figure 2 shows the longitudinal RVS bull’s eye plots with median strain values of both cardiomyopathies adapted from LVS pattern. The typical CA apical sparing pattern of the LV was observed in the septal region, but not detected in the RV free wall. In FD patients, the septal region showed a more reduced RVS than the RV free wall. RVS of the basal to mid ventricular region and the apical septal region was significantly reduced in CA compared to FD patients (*p* ≤ 0.001), while free wall apical RVS presented no significant difference (*p* = 0.474). The ratio of the basal and mid ventricular septal region to the basal and mid ventricular free wall region was significant lower in CA patients. An impaired RV function, as defined by global RVS > − 20% and/ or free wall RVS > − 23%^30,31^, was prevalent in 64 CA (96%) and 28 FD patients (72%). Nevertheless, only 44 CA (50%) and 11 FD patients (23%) presented a reduced RV function, as measured by standard echocardiographic parameters (TAPSE < 16 mm, RV-Sʹ < 10 cm/s and/ or RVFAC < 35%^24^).

**Figure 3:**
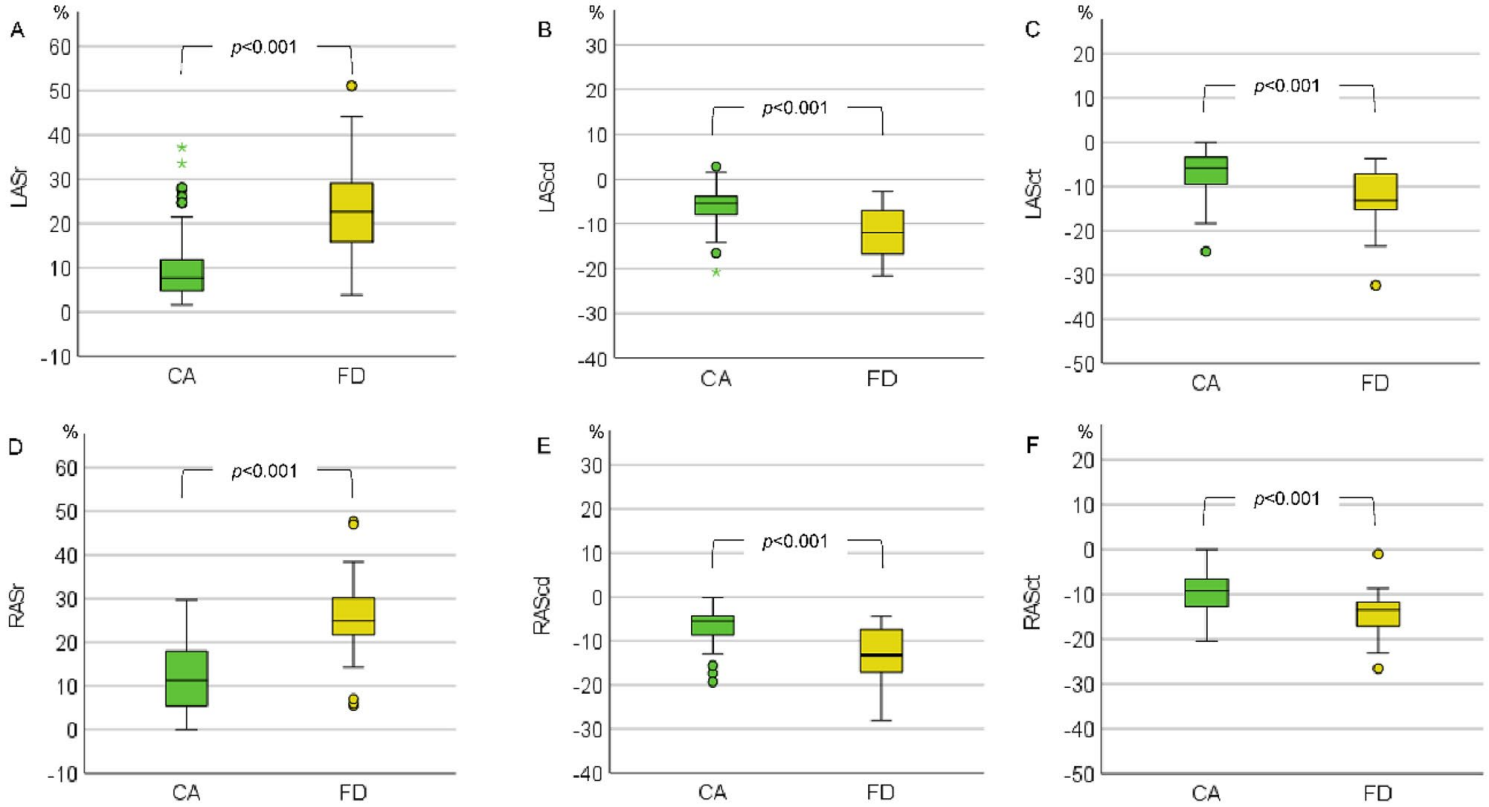
Left (**A**–**C**) and right atrial strain (**D**–**F**) in cardiac amyloidosis (CA) and Fabry disease (FD). LASr, left atrial reservoir strain; LAScd, left atrial conduit strain; LASct, left atrial contraction strain; RASr, right atrial reservoir strain; RAScd, right atrial conduit strain; RASct, right atrial contraction strain. ○) The dot indicates the exceeding of the 1.5-fold interquartile range (IQR). *) The star indicates the exc2eeding of the threefold IQR:

LAS and RAS including ASr, AScd and ASct were analysed in 82 CA and 43 FD patients as well as in 70 CA and 40 FD patients, respectively. Atrial strain parameters were impaired in both groups, with CA patients demonstrating significantly lower LAS and RAS values (Fig. 3).

### Diagnostic value of echocardiographic parameters

**Figure 4:**
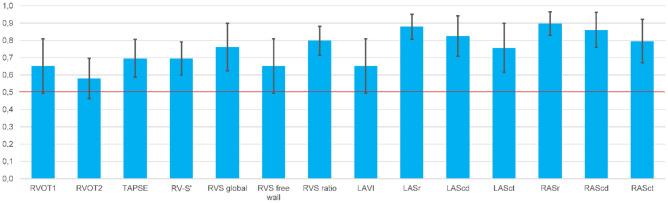
Receiver operating characteristic (ROC) curve analysis with area under the curve (AUC, blue bar) and confidence interval (CI, black line) of the following parameters to discriminate cardiac amyloidosis (CA) from Fabry disease (FD): right ventricular outflow tract (RVOT 1 and 2*), tricuspid annular plane systolic excursion (TAPSE*), systolic tricuspid annular velocity (RV-Sʹ*), right ventricular strain (RVS), right ventricular strain (RVS) ratio (ratio of basal and mid ventricular septal right ventricular strain to basal and mid ventricular free wall right ventricular strain), left atrial volume index (LAVI), left atrial reservoir strain (LASr*), left atrial conduit strain (LAScd), left atrial contraction strain (LASct), right atrial reservoir strain (RASr*), right atrial conduit strain (RAScd) and right atrial contraction strain (RASct). *) CA predicted by a smaller value. The red line marks an AUC of 0.5.

LASr and RASr showed the highest diagnostic accuracy of all evaluated atrial parameters to discriminate both cardiomyopathies (Fig. 4). A LASr and RASr value of 20% corresponded to a sensitivity of 90% and 87% and a specificity of 64% and 83% for the diagnosis of CA. A LAScd and RAScd cut off value of − 8% had a sensitivity of 67% and 63% and a specificity of 79% and 75% for the diagnosis of CA. Global RVS and RVS ratio reached the highest diagnostic accuracy of RV parameters to distinguish CA from FD. The RV ratio value of 0.4 reached a high specificity (90%) but a low sensitivity (44%) to discriminate CA.

**Figure 5:**
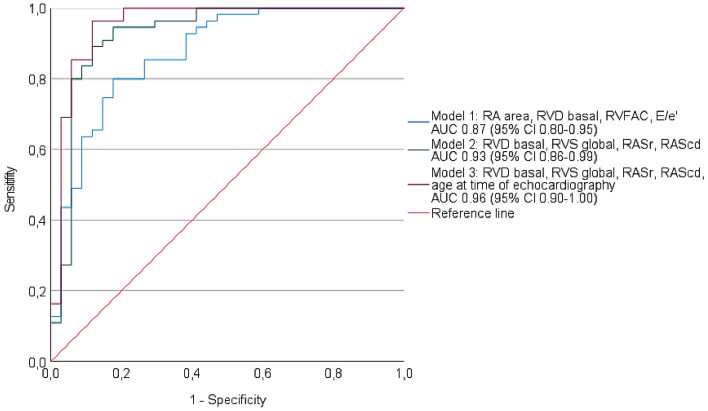
Receiver operating characteristic (ROC) curve analysis of the following variables to differentiate cardiac amyloidosis (CA) and Fabry disease (FD): Model 1 (combined variable including right atrial [RA] area, basal right ventricular [RV] diameter, right ventricular fractional area change [RVFAC], and E/eʹ; blue line), model 2 (combined variable including basal RV diameter, global longitudinal right ventricular strain [RVS], right atrial reservoir strain [RASr], and right atrial conduit strain [RAScd]; green line) and model 3 (combined variable including basal RV diameter, global longitudinal RVS, RASr, RAScd, and age at time of echocardiography; pink line). Reference line in red.

All predicters for the diagnosis of CA are listed in Table S2. Based on the binary logistic regression analysis and on the clinical relevance of the different parameters, we built the following models: We selected the four standard echocardiographic parameters RA area, basal RV diameter, RVFAC, and E/eʹ and the four parameters basal RV diameter, global longitudinal RVS, RASr, and RAScd for two different models (Fig. 5). The second model with 2D STE parameters showed a higher diagnostic accuracy compared to the first model with standard echocardiographic parameters only. For a third model, we included the age at time of echocardiography in our analysis with basal RV diameter, global longitudinal RVS, RASr, and RAScd corresponding to the highest diagnostic accuracy of 0.96 (95% CI 0.90–1.00).

## Discussion

To the best of our knowledge, the present study is the first investigation to compare the diagnostic value of an extended echocardiographic assessment of structural and functional RV, RA, and LA parameters to discriminate CA and FD. Moreover, we introduced a new diagnostic marker, the RVS ratio, to distinguish between CA and FD. AScd and ASr of both atria as well as global RVS had the highest diagnostic accuracy of the assessed parameters to distinguish CA from FD. However, the best diagnostic accuracy was achieved with a combination of 2D STE and standard echocardiographic parameters also including patient age at time of echocardiography.

A reduced RV function is well known in both cardiomyopathies. Chacko et al. reported a decrease of longitudinal RV function as assessed with RV-Sʹ, TAPSE and RVS during a follow-up period of 24 months in ATTR CA^6^. In comparison of different CA subtypes, patients with AL CA presented a more severe RV dysfunction and an impaired RV apical-to-base ratio (ratio of apical free wall RVS and the sum of basal and mid free wall RVS)^32^. In contrast, Bodez et al.^33^ did not find an apical-to-base ratio of the RVS in patients with AL and ATTR CA. Consistent with these results, we also did not detect an STE-based apical sparing pattern of the RV free wall, but only of the septum, possibly due to the small number of AL CA patients. The bull’s eye plot showed a crescent-shaped loss of septal function (Fig. 2). In CA, the LV apical sparing pattern may result from the segmental distribution of amyloid mass^34^. A minor involvement of the RV compared to the LV may also explain our findings. Nevertheless, the deposition of amyloid fibrils in the RV and atrial walls may impact on myocardial function already in an early disease stage of CA^8^. Regarding FD, the typical course of disease contains an impairment of LV function, followed by a LV and RV wall thickening and finally a reduced RV function^13,35^. In line with the FD course, we observed a more reduced RVS of the septum compared to the RV free wall (Fig. 2) which correlates with the detection of fibrosis in magnetic resonance imaging^13^. In the present study, we detected a more impaired RV function in CA patients compared to FD, which was consistent with the findings of Graziani et al. who studied a cohort of AA CA and FD patients^13,35^. Other studies compared hypertrophic, FD, and CA cardiomyopathies and found a more impaired RV function in CA and FD patients^30,36,37^. The pathophysiological mechanism is not yet fully understood. The different involvement of the RV and LV may be explainable by the diverse storage pattern with secondary mechanics including hypertrophy, inflammation, and fibrosis as well as the different anatomy of the RV with two myocardial layers compared to the LV with three layers^38^. As shown in our study and by Lillo et al.^38^ RVS was more sensitive to detect RV dysfunction in FD and CA patients. In both groups of our cohort, delta RVS was in a normal range as a possible marker for the equilibrium of RV mechanical properties.

In addition to RV dysfunction, atriopathy is also described in CA and FD patients^39,40^. We detected a more dilated LA and RA as well as a more reduced LAS and RAS in CA compared to FD patients. AScd and ASr showed the highest diagnostic accuracy to differentiate CA and FD. CA patients reportedly develop an impaired LA function due to the restrictive pattern of the LV and amyloid infiltration of the LA^39^. This corresponds to a high prevalence of atrial fibrillation in CA patients^41^. Moreover, Singulane et al.^42^ observed an impaired RAS in CA patients compared to healthy controls associated with an increased mortality. In addition to its prognostic value, atrial strain assessment may be also useful in the differential diagnosis of cardiomyopathies such as CA, hypertensive heart disease and FD^8,43,44^. In a retrospective study including CA patients and patients with LV wall thickening and negative biopsy for CA, CA patients presented with the most impaired LAS^8^. Moreover, LAS was significantly reduced in FD compared to other patients with LV wall thickening including hypertrophic cardiomyopathy and borderline myocarditis^44^. Previous studies observed a reduced LAS in FD patients, most common in patients with LV wall thickening, associated with a worse symptomatic status^40,45–47^. Linhart et al.^48^ reported the accumulation of Gb3 in the LA of FD patients in histopathological studies. In accordance with these findings, enzyme replacement therapy improved LAS, but not LA volume index (LAVI) and/ or RV function^13^. A stabilized LA function measured by LAS was also detected in patients with chaperone therapy^49^. The present study is the first assessing RAS in FD patients. We observed a reduced RAS in comparison to reported normal values^50^. All in all, our results showed a more severe atriopathy in patients with CA compared to FD which may serve as a useful future tool to differentiate CA and FD.

The present study is limited by its retrospective design. Baseline characteristics differed significantly between both groups, a problem that mirrors common clinical practice since patients with CA usually are older and present with more comorbidities than FD patients. However, these parameters may also give an indication of the specific disease as shown by our logistic regression model 3. Furthermore, FD patients received more often a specific treatment at the time of echocardiography and different disease stages were pooled in both groups. Cardiac function decreases over time in both cardiomyopathies, difference of measurements will be more present in the end stage of disease^6,12^. Moreover, not all strain parameters were assessable in all patients due to lack of RV-focused echocardiographic images or insufficient quality for 2D STE. Therefore, our results should be interpreted as a first description and hypothesis-generating and should hence be confirmed in further prospective trials.

To conclude, CA patients presented a more severe cardiomyopathy including a significantly more dilated right heart and left atrium, and overall, a more reduced cardiac function in comparison to FD. The different types of substrate storage and related pathophysiological, including inflammatory, responses as well as the specific anatomy of heart chambers may result in the seen differences between both cardiomyopathies. The combination of standard and 2D STE imaging showed the best diagnostic accuracy to distinguish CA and FD. As specific treatment with paramount prognostic importance is available for both cardiomyopathies, a correct diagnosis at an early stage of the disease is crucial for patients^14–16^. Our results suggest that a comprehensive echocardiographic assessment that integrates the evaluation of RV, RA, and LA strain into the diagnostic workup is useful to discriminate CA and FD in patients with unclear left ventricular wall thickening and suspected infiltrative or storage disease.

## Supplementary Information

Supplementary Tables.

## Data Availability

The data underlying this article will be shared on request for research purposes to the corresponding author.

## Abbreviations

2D STE: 2D speckle tracking echocardiography
AL: Monoclonal immunoglobulin light chain amyloidosis
AScd: Atrial conduit strain
ASct: Atrial contraction strain
ASE: American Society of Echocardiography
ASr: Atrial reservoir strain
ATTRv: Hereditary transthyretin amyloidosis
ATTRwt: Wild type transthyretin amyloidosis
AUC: Area under the curve
CA: Cardiac amyloidosis
CI: Confidence interval
EACVI: European Association of Cardiovascular Imaging
FD: Fabry disease
Gb3: Globotriaosylceramide
GLA: α-galactosidase A
HF: Heart failure
HFpEF: Heart failure with preserved ejection fraction
ICC: Intraclass correlation coefficient
LA: Left atrium
LAS: Left atrial strain
LV: Left ventricle
RA: Right atrium
RAS: Right atrial strain
ROC: Receiver operating characteristic curve analysis
RV: Right ventricle function
RVFAC: Right ventricular fractional area change
RVOT: Right ventricular outflow tract
RVS: Right ventricular strain
RV-Sʹ: Systolic tricuspid annular velocity
TAPSE: Tricuspid plane systolic excursion
