# Supplementary material for: Right heart and left atrial strain to differentiate cardiac amyloidosis and Fabry disease
Source: Sci Rep. 2024 Jan 30;14:2445. doi: 10.1038/s41598-024-52890-y (PMC11662012; doi:10.1038/s41598-024-52890-y)
Supplement: Supplementary file 1 — Supplementary Tables. [file 41598_2024_52890_MOESM1_ESM.docx]

**Supplement**

| **Table S1: Standard echocardiographic parameters of right ventricular, right atrial and left atrial morphology and function.** | | | | |
| --- | --- | --- | --- | --- |
|  | Cardiac amyloidosis (*n*=88) | Fabry disease (*n*=47) | *p*-Value | AUC (95% CI) |
| RVOT1, mm (IQR) | 31 (27-35), *n*=84 | 28 (26-30), *n*=46 | 0.002 | 0.66 (0.53- 0.79) |
| RVOT2, mm (IQR) | 24 (22-26), *n*=82 | 25 (22-28), *n*=45 | 0.393 | 0.47 (0.34-0.61) |
| RVD basal, mm ± SD | 42±8, *n*=86 | 33±8 | <0.001 | 0.73 (0.62-0.85) |
| RVD mid, mm (IQR) | 27 (23-32), *n*=86 | 24 (20-29) | 0.039 | 0.56 (0.42-0.70) |
| RV apex-base, mm ± SD | 68±10, *n*=85 | 67±10 | 0.927 | 0.48 (0.34-0.62) |
| RV wall thickness, mm (IQR) | 8 (6-10), *n*=79 | 6 (5-8), *n*=44 | <0.001 | 0.69 (0.56-0.82) |
| RVFAC, % ± SD | 40±11, *n*=78 | 50±10 | <0.001 | 0.75 (0.66-0.84)* |
| RV-Sʹ, cm/s ± SD | 11±4, *n*=83 | 13±3, *n*=44 | 0.003 | 0.70 (0.60-0.79)* |
| TAPSE, mm ± SD | 19±5 | 21±5 | 0.004 | 0.69 (0.59-0.78)* |
| RA area, cm^2^ ± SD | 22±6, *n*=85 | 16±5 | <0.001 | 0.72 (0.60-0.83) |
| LAVI, ml (IQR) | 48 (38-58), *n*=82 | 39 (29-52), *n*=40 | 0.002 | 0.72 (0.60-0.83) |
| IAS, mm ± SD | 8±3, *n*=79 | 6±2, *n*=39 | 0.001 | 0.68 (0.55-0.81) |
| IAS thickening, n (%) | 51 (58), *n*=79 | 14 (30) | <0.001 | 0.61 (0.48-0.75) |
| E, m/s ± SD | 0.9±0.3, *n*=87 | 0.7±0.2 | <0.001 | 0.75 (0.63-0.86) |
| E/A | 1.5 (1.0-2.8), *n*=62 | 1.0 (0.8-1.5), *n*=44 | <0.001 | 0.70 (0.58-0.82) |
| E/eʹ | 16 (11-21), *n*=87 | 9 (7-12) | <0.001 | 0.84 (0.75-0.94) |
| Continuous variables are shown as mean ± standard deviation (normally distributed) or median and interquartile ranges (IQR, not normally distributed), categorical variables are given as absolute number with percentages. AUC, area under the curve to predict cardiac amyloidosis; 95% CI, 95% confidence interval; RVOT, right ventricular outflow tract; RVD, right ventricular diameter; RV apex-base, right ventricle apex to base; RV wall thickness, right ventricular wall thickness; RVFAC, right ventricular fractional area change; RV-Sʹ, systolic tricuspid annular velocity; TAPSE, tricuspid annular plane systolic excursion; RA area, right atrium area; LAVI, left atrial volume index; IAS, interatrial septum; E, E wave; A, A wave; eʹ, eʹ wave. *) CA predicted by a smaller value. | | | | |

| **Table S2:**   **Predictors for the diagnosis of cardiac amyloidosis in binary logistic regression analysis.** | |
| --- | --- |
|  | Odds ratio (OR) with 95% confidence interval (CI) |
| Age at time of diagnosis | 1.73 (1.30-2.31) |
| LVEF | 0.91 (0.86-0.95) |
| RVOT 1 | 1.11 (1.03-1.20) |
| RVD basal | 1.17 (1.10-1.24) |
| RV wall thickness | 1.26 (1.07-1.49) |
| RVFAC | 0.91 (0.87-0.95) |
| RV-Sʹ | 0.85 (0.77-0.95) |
| TAPSE | 0.90 (0.83-0.97) |
| RA area | 1.20 (1.11-1.30) |
| LAVI | 1.03 (1.00-1.05) |
| IAS | 1.36 (1.12-1.66) |
| E | 18.07 (3.28-99.71) |
| E/A | 2.71 (1.46-5.05) |
| E/eʹ | 1.20 (1.11-1.30) |
| Global longitudinal RVS | 1.32 (1.17-1.48) |
| Free wall longitudinal RVS | 1.12 (1.10-1.33) |
| LASr | 0.86 (0.82-0.91) |
| LAScd | 1.29 (1.17-1.42) |
| LASct | 1.21 (1.10-1.33) |
| RASr | 0.82 (0.76-0.89) |
| RAScd | 1.33 (1.19-1.49) |
| RASct | 1.21 (1.09-1.35) |
| LVEF, left ventricular ejection fraction; RVOT, right ventricular outflow tract; RVD, right ventricular diameter; RV wall thickness, right ventricular wall thickness; RVFAC, right ventricular fractional area change; RV-Sʹ, systolic tricuspid annular velocity; TAPSE, tricuspid annular plane systolic excursion; RA area, right atrium area; LAVI, left atrial volume index; IAS, interatrial septum; E, E wave; A, A wave; eʹ, eʹ wave; RVS, right ventricular strain; LASr, left atrial reservoir strain; LAScd, left atrial conduit strain; LASct, left atrial contraction strain; RASr, right atrial reservoir strain; RAScd, right atrial conduit strain; RASct, right atrial contraction strain. | |
